# Non-basic amino acids in the hemagglutinin proteolytic cleavage site of a European H9N2 avian influenza virus modulate virulence in turkeys

**DOI:** 10.1038/s41598-020-78210-8

**Published:** 2020-12-04

**Authors:** Claudia Blaurock, David Scheibner, Maria Landmann, Melina Vallbracht, Reiner Ulrich, Eva Böttcher-Friebertshäuser, Thomas C. Mettenleiter, Elsayed M. Abdelwhab

**Affiliations:** 1 Friedrich-Loeffler-Institut, Federal Research Institute for Animal Health, Südufer 10, 17493 Greifswald-Insel Riems, Germany; 2 Institute of Veterinary Pathology, Faculty of Veterinary Medicine, Leipzig University, An den Tierkliniken 33, 04103 Leipzig, Germany; 3 Institute of Virology, Philipps University Marburg, Hans-Meerwein-Straße 2, 35043 Marburg, Germany

**Keywords:** Evolution, Genetics, Microbiology, Molecular biology

## Abstract

H9N2 avian influenza virus (AIV) is the most widespread low pathogenic (LP) AIV in poultry and poses a serious zoonotic risk. Vaccination is used extensively to mitigate the economic impact of the virus. However, mutations were acquired after long-term circulation of H9N2 virus in poultry, particularly in the hemagglutinin (HA) proteolytic cleavage site (CS), a main virulence determinant of AIV. Compared to chickens, little is known about the genetic determinants for adaptation of H9N2 AIV to turkeys. Here, we describe 36 different CS motifs in Eurasian H9N2 viruses identified from 1966 to 2019. The European H9N2 viruses specify unique HACS with particular polymorphism by insertion of non-basic amino acids at position 319. Recombinant viruses carrying single HACS mutations resembling field viruses were constructed (designated G319, A319, N319, S319, D319 and K319). Several viruses replicated to significantly higher titers in turkey cells than in chicken cells. Serine proteases were more efficient than trypsin to support multicycle replication in mammalian cells. Mutations affected cell-to-cell spread and pH-dependent HA fusion activity. In contrast to chickens, mutations in the HACS modulated clinical signs in inoculated and co-housed turkeys. G319 exhibited the lowest virulence, however, it replicated to significantly higher titers in contact-turkeys and in vitro. Interestingly, H9N2 viruses, particularly G319, replicated in brain cells of turkeys and to a lesser extent in mammalian brain cells independent of trypsin. Therefore, the silent circulation of potentially zoonotic H9N2 viruses in poultry should be monitored carefully. These results are important for understanding the adaptation of H9N2 in poultry and replication in mammalian cells.

## Introduction

Avian influenza viruses (AIV) belong to genus Influenza A Virus (IAV) in the family *Orthomyxoviridae*. AIV are enveloped viruses with a segmented single-strand RNA genome of negative polarity. The genome is composed of eight gene segments (PB2, PB1, PA, HA, NP, NA, M, and NS) which encode more than 11 viral proteins^1^. According to the different antigenic properties of the two surface glycoproteins hemagglutinin (HA) and neuraminidase (NA), AIV are classified into 16 HA (H1—H16) and 9 NA (N1 – N9) subtypes. Each virus contains one HA and one NA subtype with possible 144 HxNy combinations^2^. Wild birds are the natural reservoir for all AIV subtypes and domestic birds acquire AIV infection after direct or indirect contact with wild birds. In poultry, H1-H16 subtypes are low pathogenic (LP), while H5 and H7 subtypes can become highly pathogenic (HP) to cause up to 100% mortality. Therefore, preventive culling of H5 and H7 infected poultry is recommended by the World Organization for Animal Health (OIE)^3^. The HA plays an important role in virus virulence and interspecies transmission^4–6^. Activation of HA by proteolytic cleavage into HA1 and HA2 subunits is important for exposing the HA2-fusion peptide, which mediates pH-dependent merge of viral and host cell membranes and subsequently the release of viral RNA into the host cell^7,8^. The HA cleavage site motifs (CS) of HPAIV specify a stretch of basic amino acids (aa) which comply with the minimum polybasic motif required for the cleavage by ubiquitous furin-like enzymes (R-X-K/R-R)^9^. LPAIV possess monobasic HACS, which is activated by trypsin-like enzymes (e.g. trypsin, airway-trypsin like enzymes, transmembrane serine proteases “TMPRSS”) which are restricted to the respiratory and digestive tracts^10,11^. Therefore, replication of LPAIV is usually limited, and morbidity and mortality are reduced compared to the HPAIV which cause systemic infections resulting in multiple organ failure and up to 100% mortality^3^.

H9N2 viruses are the most widespread AIV in poultry worldwide. They infect a wide range of birds and mammals including humans^12,13^. In birds, the virus is endemic in many countries and a number of genetic lineages are established (e.g. G1, Y280, Korean lineages)^14^. H9N2 are classified as LPAIV and some H9N2 viruses are poorly adapted to poultry, however, they may cause severe morbidity and considerable mortality in chickens even without concomitant bacterial or viral co-infections^15–17^. Although chickens and turkeys belong to Galliformes, turkeys are generally more vulnerable to AIV-induced morbidity and mortality than chickens^3^. Compared to mammals^18,19^, genetic determinants for adaptation of H9N2 in both species are not well understood. In chickens, mutations in the HA head domain of Asian-H9N2 contributed to efficient virus transmission^20^ and insertion of basic aa in the HACS increased pathogenicity of some strains^21,22^. In turkeys, molecular markers for the adaptation of H9N2 are not known.

In Europe, H9N2 outbreaks, particularly in turkeys, have been reported from Poland, UK and the Netherlands^23–25^. In Germany, recurrent outbreaks caused by Korean-like H9N2 lineage have been increasingly observed since 2012 mainly in turkeys, and autogenous vaccines have been used in some regions^26,27^. In this study, polymorphism in the HACS was determined after analysis of the HA sequences of European and non-European H9N2 viruses. Recombinant viruses were constructed and the impact of these mutations on virus fitness in vivo and in vitro in turkeys and chickens was studied.

## Materials and methods

### Sequence analysis

All HA protein sequences of European and non-European H9N2 viruses were retrieved from GenBank and GISAID until 21–01-2020. Sequences of laboratory adapted viruses and those with ambiguous amino acid sequences were deleted. The remaining sequences were aligned using Multiple Alignment using Fast Fourier Transform (MAFFT)^28^ and further analyzed using Geneious Prime. The polymorphism in position 4 (P4) to position 1 (P1) in the cleavage site (positions 317 – 320 H9 HA numbering after removal of the signal peptide sequence) was determined.

### Cells, viruses and plasmids

Human-embryonic kidney 293 T cells (HEK-293 T), Madin-Darby canine kidney cells (MDCK), MDCK type II (MDCKII) cells, bat brain cells (FLG-R, CVCL_0I97) cat brain cells (CEB1-R), warthog brain cells (PHA-B-1-R) and mouse brain cells (MDIG-1-R) were obtained from the cell-culture collection at the Veterinary Medicine of Friedrich-Loeffler-Institut (FLI), Germany. MDCK-HAT and MDCK-TMPRSS2 cells have been previously described^29^. Primary chicken embryo kidney (CEK) cells were prepared from 18–19 day-old chicken embryos and primary turkey embryo kidney (TEK) cells from 23 day-old turkey embryos^30^. Turkey embryo brain (TEB) cells were prepared from 23 day-old turkey embryos according to the standard protocols^30^. A/turkey/Germany/AR1685/2016 (H9N2) (GISAID ID: 486,439) (designated hereafter as K319) was obtained from the repository of FLI kindly provided by Timm C. Harder. All plasmids of K319 were cloned in a previous project (Mostafa et al. submitted) as previously published^31^. pCAGGS-expression plasmid was kindly provided by Stefan Finke. Cloning of the HA gene into pCAGGS was done after amplification of the HA genes from pHW-HA plasmids using specific primers containing XhoI and ClaI restriction sites (available upon request). Green fluorescence protein (GFP) pcDNA-expression plasmid was kindly provided by Barbara Klupp.

### Generation of recombinant viruses

The HA of K319 virus was modified using QuikChange II XL Site-Directed Mutagenesis Kit (Agilent Technologies, USA). Primers used for mutagenesis are available upon request. Mutagenesis reactions were treated with 10 U/µl DpnI for one hour at 37 °C. Transformation of XL Gold ultracompetent cells was performed according to the manufacturer’s instructions (Agilent Technologies, USA) and 400 µl were plated on Luria–Bertani (LB)-agar (Invitrogen, USA) supplemented with ampicillin (Roth, Germany) overnight at 37 °C. Colonies were inoculated in LB broth supplemented with ampicillin and incubated overnight in a shaker at 37 °C/300 rpm. Plasmids were extracted by QIAprep Plasmid Kits (Qiagen, Germany) and the concentration was adjusted to 1 µg/µl. Viruses were rescued in HEK293T and MDCKII co-culture using Lipofectamine 2000 and OptiMEM (Gibco, USA) as previously described^32^. Supernatants of transfected cells were inoculated into 9–11 day-old specific pathogen free (SPF) embryonated chicken eggs (ECEs) (VALO BioMedia GmbH, Germany). Eggs were examined daily for embryo activity for 5 days post-inoculation (dpi) and chilled at 4—8 °C for 1—2 days. Then, the allantoic fluid was collected under sterile conditions from each inoculated egg and the hemagglutination titer was determined using hemagglutination (HA) test according to the standard protocol^33^. Sterile allantoic fluids (after plating on sheep blood agar) with HA titer > 16 were pooled together and aliquots were stored at—70 °C until use. Virus titration of working stocks was determined by plaque test as described below. Furthermore, unwanted mutations were excluded by sequencing of plasmids and virus stocks. Sequences of all viruses in this study were generated after amplification of all gene segments using One-Step RT-PCR kit (Qiagen, Germany) and universal primers^31^ or internal primers (available upon request). Amplicons were extracted in 1% (w/v) agarose (Biozym Scientific GmbH, Germany) gels using QIAquick Gel Extraction Kit (Qiagen, Germany). Purified gene products were subjected for Sanger sequencing by an ABI BigDye Terminator v.1.1 Cycle Sequencing Kit (Applied Biosystems, Germany). Nucleotide and deduced aa sequences were analyzed using Geneious Prime.

### Replication kinetics in different cell lines

The replication of recombinant viruses in primary CEK and TEK cells as well as indicated cell lines was compared using a multiplicity of infection (MOI) of 0.001. Viruses were incubated with the indicated cells in 12-well plates at 37 °C and 5% CO_2_ for 1 h (h). The virus inoculum was removed and cells were treated with citrate buffered (pH 3.0) saline (CBS) for 2 min (min) to inactivate extracellular virions. Afterwards, cells were washed twice with phosphate buffer saline (PBS). Finally, cells (except MDCK-HAT and MDCK-TMPRSS2) were covered with minimal essential medium (MEM) containing 2.8% bovine serum albumin (BSA) (MP Biomedicals, USA) and with or without 2 µg/mL TPCK-treated trypsin (Sigma-Aldrich, USA) and incubated at 37 °C and 5% CO_2_. MDCK-HAT and MDCK-TMPRSS2 were covered with MEM containing BSA and 0.2 µg/ml Doxycycline (Sigma-Aldrich, USA) as described^29^. Cells were harvested at the indicated time points and stored at − 70 °C until use. The replication kinetics were conducted in duplicates and repeated two times for each type of cells. Virus progeny was titrated by plaque test as described below. The results are expressed as mean and standard deviation of all replicates as log_10_ plaque forming unit per ml (Log_10_ pfu/ml).

### Plaque test

Virus titration was done in MDCKII cells in 12-well plates using standard plaque assay. Briefly, viruses were ten-fold serial diluted in MEM. Confluent cells were infected for 1 h with virus dilutions at 37 °C and 5% CO_2_. The extracellular viruses were adsorbed and cells were washed twice with PBS. Cells were covered by semi-solid agar (Bacto Agar; BD, France) containing MEM, 2 µg/ml TPCK-trypsin and 4% BSA (MP Biomedicals, USA). Plates were incubated at 37 °C and 5% CO_2_ for 3 days, cells were fixed with 10% formaldehyde containing 0.1% crystal violet and were incubated at room temperature (rt) for at least 24 h. Virus titration was performed in duplicates. Virus titers were calculated after counting the number of plaques multiplied by the reciprocal of virus dilution. The final titers were calculated and expressed as Log_10_ pfu/ml. Cell-to-cell spread was determined by measuring the diameter of 100 plaques of each virus using Nikon NIS-Elements imaging software (Nikon, Düsseldorf, Germany). Plaque diameter is shown as mean and standard deviation.

### Western Blot

The impact of trypsin, HAT or TMPRSS2 on HA cleavability was investigated using standard Western Blot procedures. CEK cells were transfected with 5 µg of the different HA-pCAGGS plasmids using Lipofectamine 2000 and OptiMEM for 24 h. Moreover, CEK cells were cotransfected with 2 µg pCAGGS-HA-plasmid and 500 ng TMPRSS2 plasmid, incubated for 24 h and harvested as described below. Furthermore, MDCK-HAT cells were infected with the six different viruses using a MOI of 0.1 for 24 h at 37 °C. At 24 h post transfection or post-infection, cells and supernatant were harvested and subjected for two cycles of centrifugation at 13,000 rpm for 10 min and washing of the pellets with 1 × PBS. Finally, pellets were solved in PBS and Laemmli buffer (Serva, Germany). The samples were heated for 5 min at 95 °C, then stored at -20 °C or directly used for detection of viral proteins in 12% Sodium Dodecyl Sulfate Polyacrylamide Gel Electrophoresis (SDS-PAGE). Briefly, 20 µl of each sample was transferred to a polyacrylamide gel to separate proteins with a molecular mass between 10 and 100 kDa. An electrophoresis chamber (BioRad, Germany) was filled with 1 × SDS-PAGE-buffer (10xPAGE Buffer: 0.25 M Tris, 2 M Glycin, 1% SDS), protein solutions containing SDS-sample buffer were loaded onto the gel and separated at 200 V for 45—60 min. Size of the indicated proteins was assessed against PageRuler protein ladder (Thermo Fisher, USA). Binding of viral proteins to a polyvinylidene fluoride membrane (PVDF) (GE Healthcare Life science, Germany) was done after electrotransfer at 20 V for 90 min per blot. After blocking the membrane with 5% skim milk in 1 × Tris-buffer-saline 2.5% containing 0.25% Tween20 (TBS-T) (Applichem GmbH, Germany) for 1 h at rt on a rocking platform. Serum of an infected turkey (1:100) in the current study was incubated with each membrane overnight at 4 °C. Membranes were washed by 1 × TBS-T and incubated with TBS-T containing peroxidase-labelled secondary anti-turkey antibodies (1:20,000) for 60 min at rt. Visualization of viral proteins was done by chemiluminescence using Clarity Western ECL Substrate (BioRad, Germany) Kit. Images were captured by Bio-Rad Versadoc 4000 Molecular Imager (BioRad, Germany) and Quantity One software (BioRad, Germany).

### Syncytium formation assay

Monolayers of CEK cells in a 24-well-plate were transiently transfected with 100 ng of GFP-pcDNA plasmid and 600 ng of the different pCAGGS-HA-plasmids using Lipofectamine 2000 and OptiMEM as previously done^34^. After 210 min of incubation at 37 °C, OptiMEM was exchanged to 1 ml MEM with 5% fetal calf serum (FCS) (Biowest, Germany). The transfected cells were incubated for 16 h at 37 °C. Cells were treated with 0.05% trypsin for 10 min at rt and incubated for 15 min at 37 °C with 1 ml MEM containing Earle’s balanced salts and 10% FCS. To induce membrane fusion, cells were incubated with 1 ml of PBS which was adjusted to pH 4.0, 4.2, 4.4, 4.6, 4.8, 5.0, 5.2, 5.2, 5.4, 5.6, 5.8 or 6.0 with HCl, for 4 min at rt. Cells were washed twice with 1xPBS and were then incubated with MEM containing 10% FCS for 4 h and finally fixed with 4% PFA. The number and the area of green-fluorescing syncytia with three or more nuclei within 10 fields of view (5.5 mm^2^ each) using an Eclipse Ti-S Fluorescence microscope and the NIS-Elements software (Nikon) as described^34^. The area of syncytia was divided by the number of syncytia to determine the average syncytia area. The larger the average area of syncytia, the higher the fusion activity.

## Animal experiment

### Ethical statement

The animal experiments were carried out in the experimental animal facilities of the FLI, Germany following the German Regulations for Animal Welfare. All experiments were approved by the authorized ethics committee of the State Office of Agriculture, Food Safety, and Fishery in Mecklenburg – Western Pomerania (LALLF M-V; permission number 7221.3–1.1–051-12) and the commissioner for animal welfare at the FLI representing the Institutional Animal Care and Use Committee (IACUCs).

### Experimental design

SPF ECE were incubated in the hatchery facilities at the FLI for 21 days and one-day old turkeys were purchased from commercial source. The turkey poults were tested to exclude bacterial (i.e. Salmonella, E.coli, ORT), viral (i.e. AIV, NDV, TRT) and protozoal (i.e. Coccidia) infections. All birds received water and feed ad-libitum. Six week-old chickens or turkeys were allocated to different groups of 15 birds each in separate animal rooms. Ten birds were inoculated with each recombinant virus oculonasally (ON) with 10^5.7^pfu/bird. At 1 day post inoculation (dpi), 5 sentinel chickens or turkeys were added to assess chicken-to-chicken or turkey-to-turkey transmission. Furthermore, 10 chickens were intravenously (IV) inoculated with selected viruses to determine the intravenous pathogenicity index (IVPI). A negative control group was left un-inoculated. All birds were observed daily for 10 days. Oropharyngeal (OR) and cloacal (CL) swabs were collected at 4 dpi using serum-free MEM containing antibiotics (in 1 L: 5.6 ml BSA, 1% enrofloxacin (Bayer, Germany), 0.5% lincomycin (WDT, Germany), 0.1% gentamycin (aniMedica GmbH, Germany). Swabs were frozen at − 70 °C until use. At 4 dpi, 3 birds per group were slaughtered and organ samples from nare, trachea, lung, airsac, pancreas, duodenum, kidney, thymus, bursa and brain were used for virus titration and/or histopathological examination. At the end of the experiment, all birds were humanely killed under deep anesthesia using Isoflurane (CP-Pharma, Germany).

### Detection of viral RNA

RNA was extracted from swabs and organ samples using NucleoMagVet 8/96 PCR Clean-up Core Kit (Macherey & Nagel GmbH, Germany) in KingFisher Flex Purification System (Thermo Fisher Scientific, USA). The amount of viral RNA was determined in different samples using SuperScript III Platinum One-Step qRT-PCR Kit (Invitrogen, Germany). A generic quantitative real-time polymerase chain reaction (RT-qPCR) targeting partial AIV-M gene sequence^35^ was performed in AriaMx Real-time PCR System (Agilent, Germany). Standard curves were run in each RT-qPCR plate using ten-fold serial dilutions of K319 virus (10^1^ to 10^5^ pfu/ml). The relative amount of viral RNA was quantified by plotting the Ct-values in the standard curves and results are expressed as mean and standard deviation (equivalent Log_10_ pfu/ml). Moreover, selected samples were inoculated in SPF ECE for virus isolation as recommended^36^. Furthermore, virus excreted in oropharyngeal (OR) and cloacal (CL) swabs from inoculated and contact birds was subjected for Sanger sequencing.

### Seroconversion

Blood samples were collected at the end of the animal experiment from all chickens and turkeys, and serum was separated after 24 h incubation in the fridge. Sera were tested for anti-AIV nucleoprotein (NP) using enzyme-linked immunosorbent assay (ELISA) by ID screen Influenza A Antibody Competition Multispecies kit (IDvet, France) and plates were read using Tecan ELISA reader. According to the manufacture guideline samples below 45% were considered negative.

### Histopathology and immunohistochemistry

Histopathological lesions and distribution of AIV matrixprotein (MP) antigen in different organs were studied by histopathological and immunohistochemical (IHC) techniques. Samples were embedded in paraffin wax and sectioned at 2 – 4 µm. For histopathological examination, slides were stained with hematoxylin and eosin and the severity of lesions was determined using an ordinal scale: 0 = no; 1 = mild; 2 = moderate, and 3 = severe necrosis and/or necrotizing inflammation. Furthermore, for immunohistochemical examination slides were stained using the avidin–biotin–peroxidase complex method (Vectastain PK 6100, Vector Laboratories Burlingame, USA) with citric buffer pretreatment, a primary monoclonal mouse anti-MP antibody (M1Hb-64, 1:100), and a secondary biotinylated anti-mouse IgG (BA 9200, Vector Laboratories Burlingame, USA) antibody (1:200) in IHC as described^37^. The distribution of parenchymal and endothelial MP antigen in different organs was semi-quantitatively scored, each on an ordinal scale: 0 = negative; 1 = focal or oligofocal, 2 = multifocal, and 3 = coalescing to diffuse antigen.

### Statistics

Data were analyzed using GraphPad Prism version 8.1.0 and differences were considered significant at a *p* value of *p* < 0.05. Plaque diameter was analyzed with Kruskal–Wallis with Dunn’s test. Replication kinetics in different cell lines were analyzed using one-way ANOVA with post hoc Tukey tests and Kruskal–Wallis with Dunn’s test. Viral shedding in oral swabs at 4 dpi were compared using Mann–Whitney Wilcoxon test. Results were considered significant when *p* value in both tests were < 0.05.

## Results

### European H9N2 viruses specify unique HACS motifs, mostly due to substitutions of non-basic amino acids

**Figure 1:**
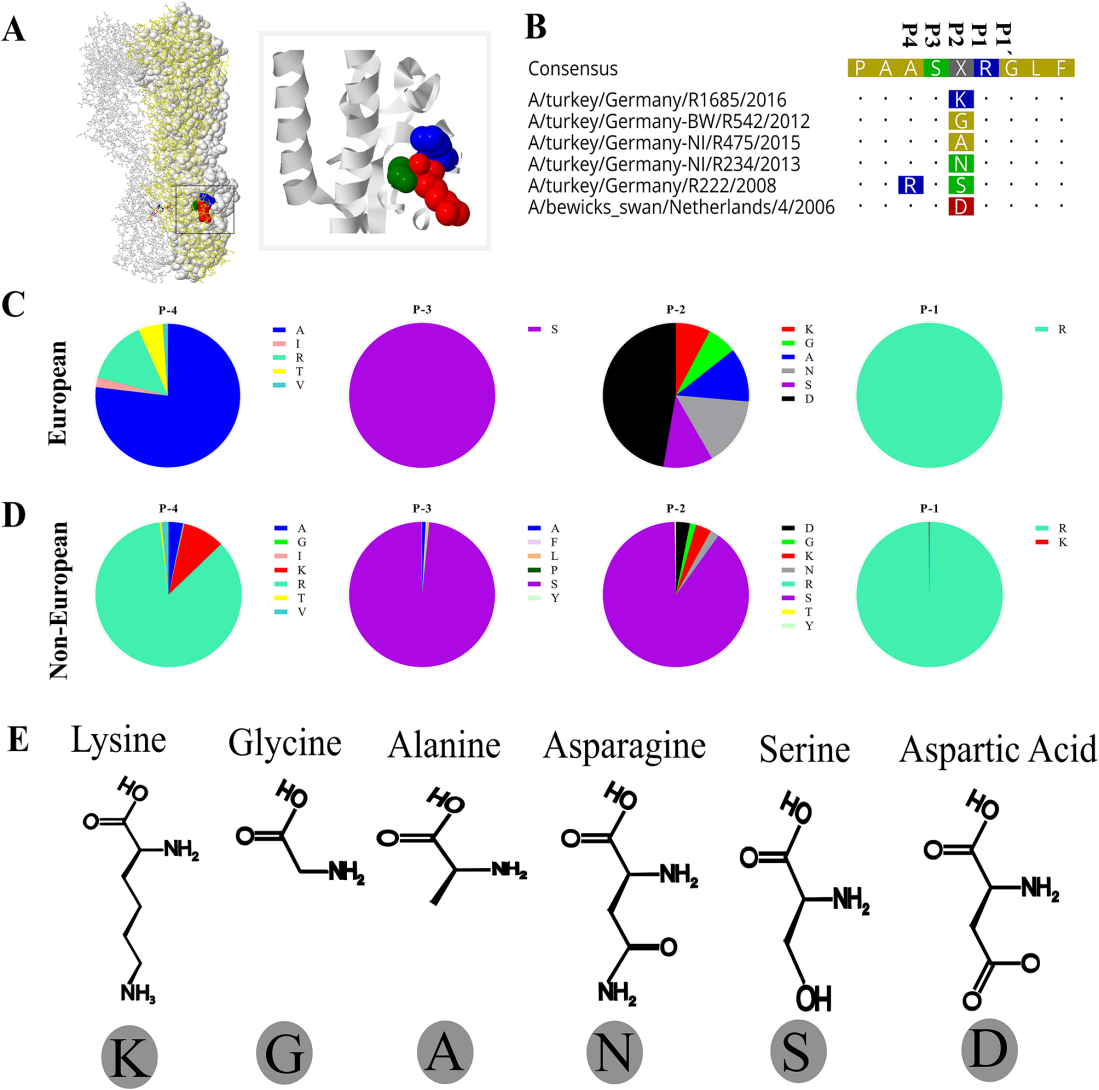
Polymorphism in the hemagglutinin cleavage site (HACS) of European and non-European H9N2 sequences. The 3D structure of H9N2-K319 HA trimer showing P2 (residue 319) in blue, arginine in P1 (red) and glycine in P1´(green) was generated by SWISS-Model and further edited by Geneious (**A**). Alignment of the cleavage site of representative European viruses showing polymorphism (K, G, A, N, S, D) in position P2 (**B**). Prevalence of polymorphism in the HACS of European H9N2 (n = 82) (**C**) and Non-European H9N2 (n = 2926) (**D**) in H9N2-HA sequences retrieved from GISAID and GenBank on 20–01-2020. All sequences specifying S contains R in position P4. Positions P1 and P3 are highly conserved, while position P2 was more variable than P4, particularly in the European-H9N2 sequences (C-D). Structure and size of amino acids in position P2 are shown (**E**).

Polymorphism in the cleavage sites (Fig. 1A,B) from position P4 to position P1 (residue 317 to 320 in H9 numbering) in European (n = 82) and non-European (n = 2926) H9N2 viruses from 1966 to 2019 were analyzed (Fig. 1C,D; Table 1, Supplementary Table S1). Sequences of the European viruses represent 21 turkey, 15 chicken and 46 wild bird isolates. In total, 36 different HACS motifs were identified (P4-P1 only). The European viruses had 12 different motifs and the non-European viruses exhibited 32 different HACS motifs (Table 1). Four HACS motifs were observed only in the European and 24 only in the non-European viruses (Table 1). While the majority of non-European H9N2 possessed RSSR/G (81.5%), KSSR/G (5.9%) or KSKR/G (3.3%) in the HACS, the European viruses specified different motifs due to accumulation of non-basic aa including ASDR/G (47.6%), ASNR/G (13.4%), ASAR/G (7.3%) or RSSR/G (11.0%). Only 4 out of 36 HACS motifs (motifs #6, 7, 27 and 35) contain dibasic or multibasic HACS. Motif #6 (AS**KR**/G) was seen in 4 European H9N2 sequences and motif #7 (**R**S**KR**/G) in 1 and 6 European and non-European H9N2 sequences, respectively. Moreover, the non-European viruses have variations in all four positions. Conversely, serine (S) in P3 and arginine (R) in P1 in the European viruses were highly conserved (100%), while P4 and P2 specified 5 (A, I, R, T, V) and 6 (G, A, N, S, D, K) different aa, respectively (Fig. 1B). At P2, the European viruses had G (6.1%), A (7.3%), N (15.9%), S (11.0%), D (50.0%) or K (9.8%), while the prevalence rate of these aa in non-European viruses was 1.4, 0.0, 1.7, 89.7, 3.1 and 3.5%, respectively. These results indicate that the HACS sequences of European H9N2 viruses differ from the non-European viruses. While serine at P2 dominated the non-European H9N2, the European H9N2 viruses had relatively comparable prevalence of G, A, N, S and K (6.1 to 15.9%), while D had a prevalence of 50.0%. G is the smallest and K is the largest aa (Fig. 1E). Since mutation (i.e. to tyrosine (Y)) at P2 affected cleavability of non-European H9N2 viruses in cell culture^38^ and replication of WSN/H1N1 in the brain of mice^39^, we decided to study the impact of non-basic aa in P2 (hereafter referred to 319) in the European H9N2 viruses in vitro and in vivo.

**Table 1:** Cleavage site motifs of European and Non-European H9N2 viruses from 1966 to 2019.

	Motif (P1/P1´)	European H9N2 (total 91)		Non-European H9N2 (total 2926)	
		No	%	No	%
1	AS**G**R/G	2	2.4	13	0.4
2	AS**A**R/G	6	7.3	0	0.0
3	AS**N**R/G	11	13.4	3	0.1
4	RS**S**R/G	9	11.0	2384	81.5
5	AS**D**R/G	39	47.6	70	2.4
6	AS**K**R/G	4	4.9	0	0.0
7	RSKR/G	3	3.7	6	0.2
8	VSDR/G	1	1.2	20	0.7
9	TSNR/G	2	2.4	0	0.0
10	TSGR/G	3	3.7	16	0.5
11	ISGR/G	1	1.2	2	0.1
12	ISDR/G	1	1.2	0	0.0
13	RSGR/G			3	0.1
14	RSNR/G			35	1.2
15	ASDK/G			1	0.0
16	VSNR/G			7	0.2
17	VSGR/G			7	0.2
18	VSSR/G			9	0.3
19	ASYR/G			7	0.2
20	ISNR/G			3	0.1
21	KSSR/G			172	5.9
22	ISSR/G			1	0.0
23	KASR/G			2	0.1
24	RYSR/G			2	0.1
25	RASR/G			21	0.7
26	RLSR/G			13	0.4
27	RSRR/G			7	0.2
28	GSSR/G			3	0.1
29	RPSR/G			2	0.1
30	RFSR/G			9	0.3
31	RSTR/G			2	0.1
32	RSSK/G			4	0.1
33	RCSR/G			1	0.0
34	RSIR/G			1	0.0
35	KSKR/G			96	3.3
36	RSNK/G			1	0.0
	Total	82	100.0	2926*	100.0

A total of 2926 non-European viruses were retrieved from GISAID and GenBank on 21–01-2020. Three sequences from chickens (2 from Iran in 2007 and 1 from Egypt in 2013) had RSNR/**R**, RSNK/**R** and KSSR/**A** motifs assuming wrong or unusual sequences in the HA2 (i.e. underlined R or A). Only motifs No. 6, 7, 27 and 35 (highlighted in grey) encode dibasic or multibasic HACS. Using reverse genetics, we generated six recombinant viruses with polymorphism at position P2 resembling the first 6 motifs (no. 1 to 6).

### Generation of six recombinant viruses with variable HACS motifs

To get an insight into the impact of non-basic aa variation at P2, six recombinant viruses were generated. In addition to the wild type K319 virus, five mutants carrying G, A, N, S or D at position 319 (designated G319, A319, N319, S319 and D319, respectively), resembling the field viruses (Fig. 1C), were rescued. Viruses were propagated in SPF ECE and the virus titers ranged from 10^5.7^ (G319) to 10^6.2^ (S319) pfu/ml (data not shown).

### Variable replication of recombinant viruses in primary turkey and chicken cells in the presence or absence of trypsin

**Figure 2:**
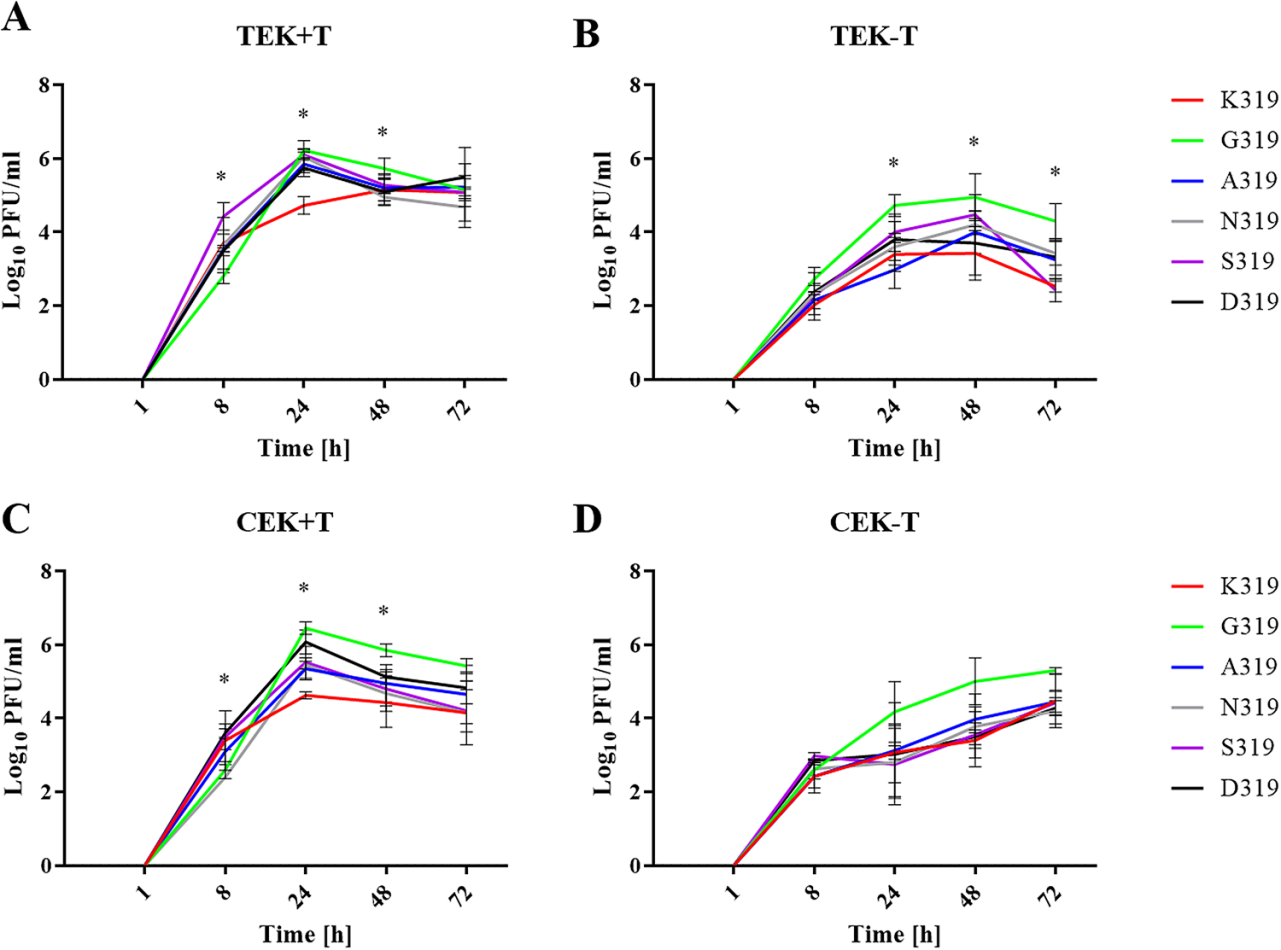
Virus replication in cell culture in the presence or absence of trypsin. Shown are the replication kinetics of the recombinant viruses 1, 8, 24, 48 and 72 h post inoculation (hpi) at an MOI of 0.001 in turkey embryo kidney (TEK) cells (**A**,**B**) and chicken embryo kidney (CEK) cells (**C**,**D**) with (+ T) and without trypsin (-T). Titers are expressed as PFU/ml and are shown as the mean and the standard deviation. The kinetics were done twice in duplicates for each cell types and data were analyzed using one-way ANOVA with post hoc Tukey tests and Kruskal–Wallis with Dunn’s test. The results were considered significant when *p* value in both tests were < 0.05 as indicated by asterisk and explained in the main text.

Replication of the recombinant viruses was studied in TEK and CEK in the presence and absence of trypsin for 1, 8, 24, 48 and 72 hpi, and virus titers were determined by plaque test in MDCKII cells (Fig. 2A–D). In TEK cells, in the presence of trypsin, the peak of virus replication was reached 24 hpi, except for K319 at 48 hpi. G319 replicated at significantly lower levels than S319 at 8 hpi (*p* < 0.002), while G319 and S319 replicated at higher levels than K319 at 24 hpi (*p* < 0.04). At 48 hpi, G319 replicated at higher levels than N319 (*p* < 0.04) and all viruses replicated at similar levels at 72 hpi (Fig. 2A). Without trypsin, all viruses replicated at comparable levels at 8 and 48 hpi and reached the peak of replication at 48 hpi. G319 replicated at significantly higher levels than A319 at 24 hpi and higher than K319 and S319 at 72 hpi (*p* < 0.006) (Fig. 2B). Although trypsin increased virus titers in TEK cells compared to cells without trypsin, only significant differences were obtained for S319 at 8 and 24 hpi (*p* < 0.04). In CEK cells, in the presence of trypsin, peak of virus replication was reached 24 hpi. G319 replicated at significantly higher titers than K319 at 24 and 48 hpi (*p* < 0.03) (Fig. 2C). D319 replicated at significantly higher titers than N319 at 8 hpi and higher than K319 at 24 hpi (*p* < 0.02) (Fig. 2C). Without trypsin, virus titers were comparably lower than in the presence of trypsin, although it was not statistically significant (p > 0.052) and peak of virus replication was delayed to 72 hpi (Fig. 2D). Comparing TEK and CEK, in the presence of trypsin, N319 replicates significantly higher in TEK than in CEK cells at 8 hpi (*p* < 0.003) (Fig. 2A,C). Without trypsin, only K319, A319 and S319 replicated at 72 hpi to significantly higher levels in TEK cells than in CEK cells (Fig. 2B,D). Taken together, all recombinant viruses replicated to higher levels and reach their maximum titer in primary turkey and chicken cells faster in the presence of trypsin. Non-basic amino acids, particularly G319, supported rapid and higher replication of H9N2 in turkey cells. Regardless of their different physiochemical properties, mutations in the HACS enhanced virus replication in TEK than in CEK. These results indicate that mutations in P2 affect virus replication in avian cells.

### TMPRSS2 and to a lesser extent HAT can support multiple-cycle replication of viruses with non-basic amino acids in the HACS

**Figure 3:**
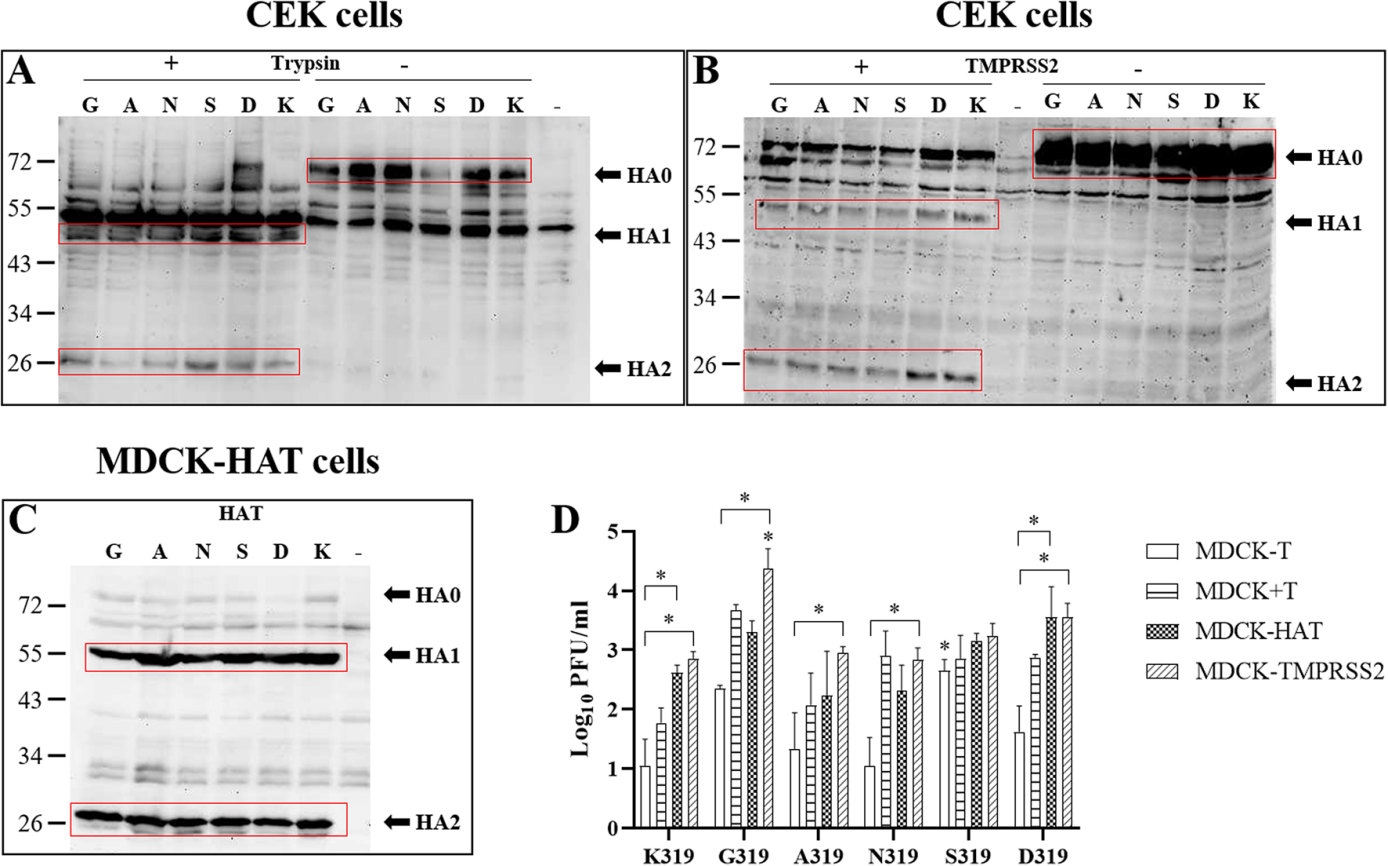
HA proteolytic activation and replication of recombinant viruses in different MDCK cell lines. Cleavage-activation of the HA of recombinant viruses carrying single mutations in the HACS in CEK cells with ( +) or without ( +) trypsin (**A**), in CEK after transfection with TMPRSS2 ( +) or left untransfected (-) (**B**) or MDCK cells expressing HAT (**C**). Western Blot figures were acquired by Quantity One Software Version 4.4 (Biorad, Germany) (https://www.bio-rad.com/webroot/web/pdf/lsr/literature/4000126-14A.pdf). Multiple cycle replication after infection of MDCK with or without trypsin, MDCK-TMPRSS2 or MDCK-HAT with different recombinant viruses at MOI of 1 for 24 h. Titers were determined by plaque assay in MDCKII cells in the presence of trypsin. Titers are expressed as PFU/ml and are shown as the mean and the standard deviation. The kinetics were done twice in duplicates for each cell types (**D**). Data were analyzed using one-way ANOVA with post hoc Tukey tests and Kruskal–Wallis with Dunn’s test. Asterisk indicates significant differences compared to K319 (*p* < 0.05).

Using Western blot, the HA of all viruses was shown to be cleaved by trypsin, TMPRSS2 and HAT whereas no cleavability was observed in the absence of these proteases (Fig. 3A–C). The proteolytic activation of HAT and TMPRSS2 to support multiple-cycle replication was studied in different MDCK cells for 24 hpi. In MDCK cells which do not produce endogenous HAT or TMPRSS2, virus titers were generally lower than in MDCK-TMPRSS2 and MDCK-HAT cells (Fig. 3D). Without trypsin, G319 and S319 replicated to higher levels than K319, A319 and N319. S319 replicated at significantly higher titers than D319 (*p* < 0.02) (Fig. 3D). In the presence of trypsin, the titer of G319 was higher than A319 and K319. In MDCK-TMPRSS2 cells, all viruses replicated at comparable levels (p > 0.2), except G319 which replicated at significantly higher titer than other viruses (*p* < 0.015) (Fig. 3D). In MDCK-HAT cells, all viruses replicated at comparable levels (p > 0.1) (Fig. 3D). Viruses replicated at higher titers in MDCK-TMPRSS2 than in MDCK-HAT cells, but it was not statistically significant (p > 0.9). All viruses except S319 replicated at significantly higher titers in MDCK-TMPRSS2 than in MDCK cells (*p* < 0.001), while only K319 and D319 replicated at significantly higher titers in MDCK-HAT than in MDCK cells (*p* < 0.05). Together, although mutations in P2 did not affect HA-cleavability by trypsin, HAT and TMPRSS2, they modulated H9N2 virus replication in a protease-dependent manner with a remarkable impact of TMPRSS2 and to a lesser extent HAT on virus replication.

### Non-basic amino acids in the HACS increased cell-to-cell spread and influenced the optimal pH-range for HA fusion activity

**Figure 4:**
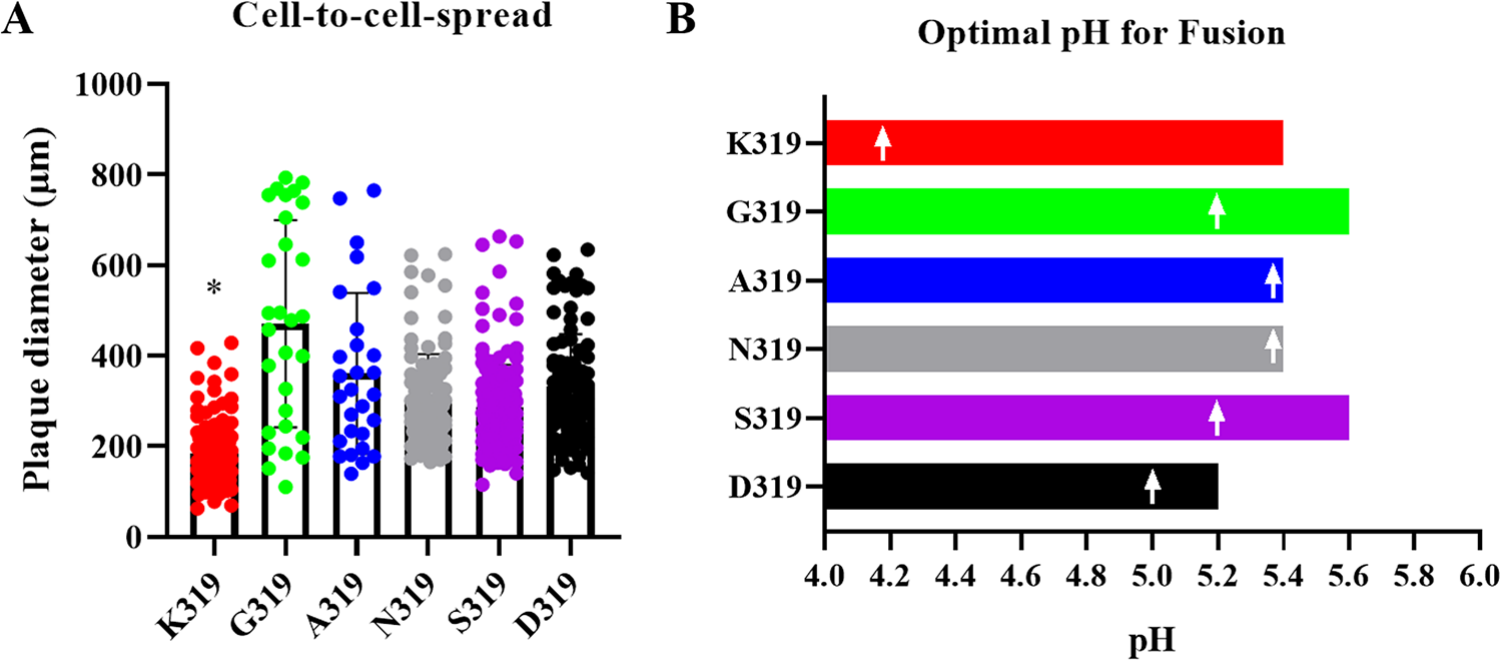
Cell-to-cell spread and pH fusion activity of recombinant viruses. Cell-to-cell spread was assessed in MDCKII cells in the presence of trypsin. Asterisk indicates that K319 induced the lowest plaque diameter compared to all other viruses (*p* < 0.05). Plaque diameter was measured by Nikon NIS Software (Nikon, Germany) (https://www.microscope.healthcare.nikon.com/de_EU/products/software/nis-elements) and analyzed with Kruskal–Wallis with Dunn’s test (**A**). pH activation of membrane fusion was determined by treating HA-transfected chicken embryo kidney cells with pH 4.0 to 6.0 at 0.2 pH-intervals. The mean size of measured syncytia (> 50) was calculated. Shown is the range of pH values at which HA fusion activity was detectable. White arrows indicate the pH at which fusion activity was highest for each HA variant (**B**).

Cell-to-cell spread of the different virus variants was investigated by plaque assays on MDCKII cells. While K319 produced significantly smaller plaques compared to other viruses (*p* < 0.0001), largest plaque diameter was observed for G319 (*p* < 0.01) (Fig. 4A). Binding of protons in the low pH environment of the endosome serves as fusion trigger for HA. The pH-dependence and optimal pH of fusion of the different HA variants was investigated in a transient-transfection based cell–cell fusion assay. For this, CEK cells were transfected with the different pCAGGS-HA plasmids and GFP-pcDNA which was used as a marker and fusion was triggered with PBS buffer at pH 4.0 to 6.0 in 0.2 intervals. 4 h after the pH shift, syncytia were measured to determine the pH for membrane fusion. All HA variants produced syncytia at pH 4.0 to 5.2. Exposure to pH 5.4 could trigger all HA variants except D319. Only S319-HA and G319-HA produced syncytia at pH 5.6. No syncytia formation was observed by any HA at pH ≥ 5.8. The highest fusion activity was observed after exposure to pH 4.2 for K319-HA, pH 5.0 for D319-HA, pH 5.2 for G319-HA and S319-HA and pH 5.4 for A319-HA and N319-HA (Fig. 4B). These results indicate that mutations in P2 have an impact on cell-to-cell spread and pH fusion activation of H9N2.

### Mutations in the HACS did not increase H9N2 virulence or transmission in chickens

**Figure 5:**
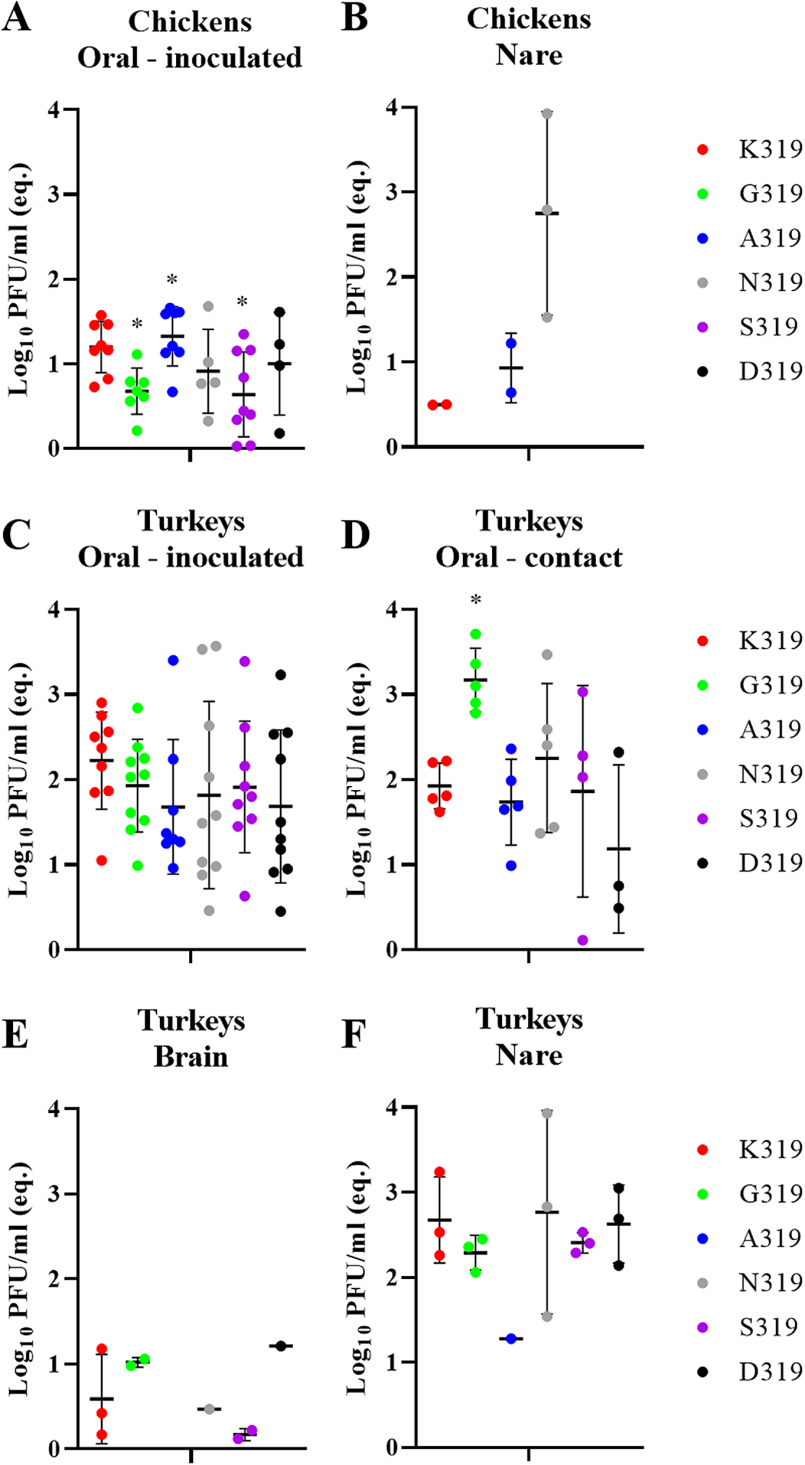
Virus detection in oropharyngeal swabs and organ samples in chickens and turkeys. Detection of viral RNA in inoculated chickens in oropharyngeal swabs (**A**) or organs (nasal cavity) (**B**) at 4 dpi was determined by RT-qPCR targeting the M gene and are expressed as equivalent Log_10_ PFU/ml. Virus excretion in oropharyngeal swabs (**C**,**D**) as well as in brain (**E**) and nasal cavity (**F**) at 4 dpi was determined by RT-qPCR targeting the M gene and are expressed as equivalent Log_10_ PFU/ml. Asterisk indicates significant difference compared to K319.

All ON or IV challenged chickens remained healthy and PI values for all viruses were 0.0, however, all birds seroconverted (Supplementary Table S2). Viral RNA of all viruses was only detected in OR swabs of inoculated chickens 4 dpi and at low titers (Fig. 5A), where RNA of K319, G319, A319, N319, S319 and D319 was detected in 8/10, 7/10, 8/10, 5/10, 9/10 and 4/10 OR swabs, respectively (Supplementary Table S2). G319 and S319 had significantly lower titers than K319 and A319 (*p* < 0.04) (Fig. 5A). Viral RNA was reported in the nare of inoculated chickens with N319 and to a lesser extent in A319- or K319-inoculated chickens (Fig. 5B). No infectious virus was obtained after direct titration of any sample in plaque test. All contact chickens remained healthy and neither antibodies nor viral RNA were detectable (Supplementary Table S2). Collectively, H9N2 used in this study is poorly transmissible in chickens and mutations in P2 have no impact on virus transmission in chickens.

### In turkeys, H9N2 exhibited higher adaptation than in chickens and some non-basic amino acids in the HACS affected virulence, replication and transmission

Direct inoculated and co-housed turkeys exhibited clinical signs including swelling of infraorbital sinus, facial edema, ruffled feather, rales and/or diarrhea. These clinical signs were observed in inoculated turkeys with N319 (10/10; PI = 0.6), K319 and D319 (9/10; PI = 0.4), S319 (3/10; PI = 0.1), A319 (2/10; PI = 0.1) and G319 (1/10; PI = 0.1) (Table 2) and in 2/5, 3/5, 4/5, 1/5, 1/5 and 1/5 co-housed turkeys, respectively (Table 2). At 4 dpi, caseous material was observed in the swollen sinuses during autopsy. Viral RNA was detected in almost all OR swabs obtained 4 dpi in inoculated turkeys and no significant differences were observed between the groups (p > 0.1) (Fig. 5C). In contact turkeys, G319 was shed in significantly higher amounts compared to K319, A319 and D319 (*p* < 0.03) (Fig. 5D). Cloacal shedding was only detected in two K319- and one A319-inoculated turkeys, although at very low titers (data not shown). In all inoculated groups, viral RNA was detectable only in the nare obtained 4 dpi and to a lesser extent in the brain (except for A319) (Fig. 5E,F). Direct titration of samples was not successful. Sequence of the HA in swab samples revealed no changes in the HACS and no additional mutations in the HA except G319 which an additional G61D mutation (H9 numbering) in inoculated turkeys. All ON-inoculated and contact turkeys seroconverted, except for one contact turkey inoculated with S319 (Table 2). Using IHC, MP antigen was detected in the nare of all inoculated turkeys, mainly in the epithelial cells of nasal chambers, infraorbital sinus and nasal glands. K319 and N319 were detected at slightly higher levels than others. MP antigen was neither detected in other examined organs nor in the endothelial cells. Histopathological changes in the nare were mostly multifocal, acute to sub-acute, lymphocytic and purulent, partially necrotizing sinusitis, rhinitis and inflammation of the nasal glands with associated edema of surrounding tissue. The inflammation caused by K319 and N319 was slightly more severe than other viruses. In the brain, mild multifocal lymphocytic perivascular infiltration was observed in turkeys inoculated with D319, A319 and S319. In the kidneys, focal interstitial lymphocytic infiltration was observed in one turkey inoculated with A319. Together, turkeys succumbed to H9N2 infection more severely than chickens. Mutations in P2 in the HACS affected virulence in turkeys.

**Table 2:** Clinical examination of turkeys after oculonasal inoculation.

	PI	Inoculated Turkeys			Contact Turkeys		
		Morbidity	Shedding	Seroconversion	Morbidity	Shedding	Seroconversion
K319	0.4	9/10*	9/10	7/7	3/5	5/5	5/5
K319G	0.1	1/10	10/10	7/7	1/5	5/5	5/5
K319A	0.1	2/10	8/10	7/7	1/5	5/5	5/5
K319N	0.6	10/10	10/10	7/7	2/5	5/5	5/5
K319S	0.1	3/10	9/10	7/7	1/5	4/5	4/5
K319D	0.4	9/10	10/10	7/7	4/5	3/5	5/5

* Number of positive birds/total examined.

Turkeys were challenged with 10^5^^.^^7^ pfu/bird and 1 dpi 5 birds were added to assess transmission. At 4 dpi, 3 directly-inoculated turkeys were euthanized to assess virus distribution and lesions in different organs. Seroconversion (number of positive birds / total examined) was tested at 10 dpi using ELISA. Clinical scoring was conducted as recommended by the OIE on a scale 0 to 3: 0 = apparently healthy, 1 = birds showed 1 clinical sign (ruffled feather, respiratory disorders, diarrhea), 2 = birds showed more than 1 clinical signs and 3 = dead birds. The pathogenicity index (PI) is the mean of all clinical scores for all inoculated birds in 10 day-observation period. Clinical examination was done blindly by two veterinarians. Shedding was determined by RT-qPCR.

### HACS mutations have an effect on virus replication in different brain cells in the presence or absence of trypsin

**Figure 6:**
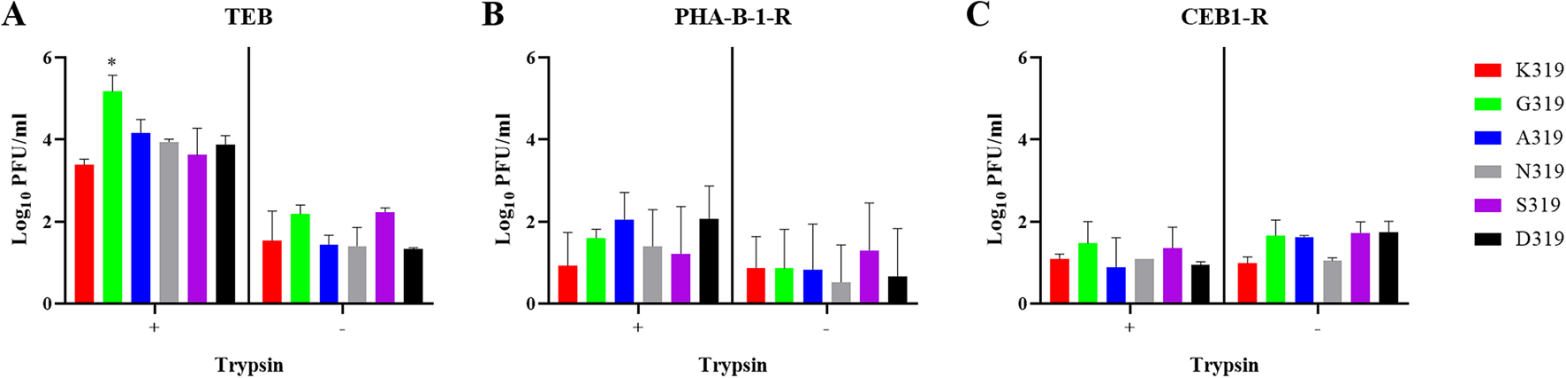
Replication of recombinant viruses in brain cells of turkeys, pigs and cats. Replication of indicated viruses was tested in primary turkey embryo brain (TEB) cells (**A**), Warthog brain PHA-B-1-R cell line (**B**) and Cat brain CEB1-R cell line (**C**) after infection with recombinant viruses at an MOI of 0.001 for 24 h in the presence ( +) or absence (-) of trypsin**.** Virus titers are expressed as log10 PFU/ml and are shown as the mean and standard deviation of three independent rounds. Asterisk indicate significant differences (*p* < 0.05) compared to wt-H9N2 K319.

Because low amounts of H9N2 RNA and mild lesions were observed in the brain of turkeys, multiple-cycle replication efficiency in brain cells obtained from different species was studied. All recombinant viruses replicated in TEB, PHA-B-1-R and CEB1-R cells independent of trypsin at an MOI of 0.001 for 24 h (Fig. 6A–C). In TEB, the addition of trypsin significantly increased virus replication and G319 revealed the highest titers (*p* < 0.01) (Fig. 6A). In PHA-B-1-R and CEB1-R cells, viruses replicated at significantly lower titers than in TEB without significant impact of trypsin in virus replication (Fig. 6B,C). Moreover, G319 was able to replicate in bat and mouse brain cells independent of trypsin, although at lower titers when compared to PHA-B-1-R and CEB1-R cells (data not shown). These data indicated that the H9N2 virus used in this study can infect brain cells of turkeys and mammals independent of trypsin, and mutations in the HACS can affect multiple-cycle replication of the virus in turkey brain cells.

## Discussion

The wide distribution of H9N2 affects the poultry industry worldwide. The virus is endemic in chickens and turkeys in several African and Asian countries, and recurrent outbreaks have been reported in Europe. European H9N2 viruses analyzed in this study had HACS motifs, which are different from the non-European H9N2 viruses. Compared to the Asian H9N2 G1-like lineage, little is known about the virulence of European H9N2 in chickens and turkeys^40,41^. Several studies have shown that non-European H9N2 viruses can increase in virulence after the insertion of basic aa in the HACS with or without reassortment with HPAIV H5N1^21,22^. Our analysis showed that the European H9N2 virus acquired several non-basic amino acids particularly in position P2 (residue 319 H9 numbering). Some of these alterations have been previously reported in Polish H9N2 viruses^42^. While amino acids at P3 and P1 are highly conserved, the variation in position 2 is remarkable. Six different amino acids were observed in European H9N2 viruses (G, A, N, S, D, K) while three were seen in the non-European viruses (Y, T, I). These results indicate a preferential selection for accumulation of non-basic amino acids in non-European viruses at this position. The inserted non-basic amino acids at P2 have different physiochemical properties, including size and polarity. These different characteristics may have an impact on the HACS conformation which could result in increased accessibility for certain proteases and thus enhanced HA cleavability, and/or the degree of exposure of the fusion peptide, resulting in the observed enhanced cell-to-cell spread (Fig. 4A) and altered optimal pH-range to trigger HA fusion (Fig. B).

Viruses used in this study replicated in different cultured cells without trypsin, although cells were infected with low multiplicity and extracellular virions were removed by treatment of cells with citrate buffered saline and washing with PBS. The efficient replication of some H9N2 viruses in primary chicken cells without exogenous trypsin has been previously reported^43^. Our results showed that TMPRSS2 and to a lesser extent HAT can support multiple-cycle replication of different viruses, particularly G319, better than trypsin. Several studies have also shown that S, R or K in P2 can affect cleavage activation of the R-S-X-R motifs in non-European H9N2 viruses by different proteases (i.e. matriptase, HAT, TMPRSS2 and furin)^11,44^. Similarly, a tyrosine at P2 affected cleavability of non-European H9N2 viruses in cell culture^38^.

Cleavage of the fusion-inactive HA0 by host proteases into HA1/HA2 subunits results in irreversible conformational changes, enabling HA2 to mediate low-pH dependent fusion of the viral and cellular membranes in the endosomal compartment and subsequently release of the viral RNA into the cytoplasm^6^. The pH value in the early endosome is about 6 to 6.3, 5 to 6 in the late endosome, and 4 to 5^45^ in the lysosome^46^. Thus, rapid fusion may enhance early virus replication before triggering the host-immune response or lysis of the virus particles. Nevertheless, pH stability is also important for persistence of AIV in the environment or in the acidic milieu of the upper airways. Therefore, AIV optimal ranges for pH-fusion-activation vary from 4.4 to 6.4^46,47^. Mutations in P2 affected pH-fusion activation in the range of pH 4.0 to 5.6. Although the mechanism is not fully understood, it is possible that the insertion of non-basic amino acids enhanced cleavability of the HA in the extracellular environment by HAT or intracellularly by TMPRSS2. It has been also reported that mutations in the head domain of the HA1 apart from the fusion peptide can trigger HA2 fusion activity of H9N2 at different pH values^48,49^.

Although chickens and turkeys are both galliform birds, they vary in their susceptibility to AIV. Generally, turkeys are more vulnerable to AIV-induced morbidity and mortality than chickens^3^. The virus used here is a turkey-origin virus similar to the vast majority of European viruses analyzed in this study, thus it is conceivable that adaptation to turkey-cells or turkeys was superior to chickens. Similarly, Turkey/Wisconsin/66(H9N2) caused no clinical signs in chickens, although all chickens seroconverted, while inoculated-turkeys exhibited mild depression, sinusitis and respiratory signs and almost all turkeys seroconverted^50^. Likewise, all chickens inoculated with a recent polish H9N2 virus of turkey origin from the 2013/2014 outbreak and 1 of 2 contact-chickens seroconverted^42^. Poor replication of two Dutch wild-bird-origin H9N2 viruses in chickens as indicated by lack of clinical signs, low virus titers in swabs and respiratory organs and low seroconversion has been reported^41^. Conversely, efficient replication and transmission of a chicken-adapted virus Ck/Hebei/LC/2008 H9N2 in chickens and turkeys have been reported, although cloacal excretion was not seen in turkeys^51^.

There is a gap in understanding the genetic determinants responsible for adaptation in chickens *vs.* turkeys. Interestingly, N337K (corresponding to N319K in our study) in the HACS was detected in the 6^th^ passage of a turkey-origin H9N2 isolated in Poland in 2013/2014 outbreak in chickens as low frequency variant and became predominant in the 7th passage indicating a role of this particular amino acid for virus adaptation in chickens^52^. However, in our study, neither N319 nor K319 exhibited any increased virulence or transmissibility in chickens or turkeys, and both viruses replicated in a trypsin-dependent manner at significantly higher levels in turkey cells than in chicken cells. It seems that variation in P2 alone is not sufficient for adaptation in chickens and additional mutations e.g. in HA, PA, NP and PB1^52^ or in the NA^53^ are probably required. Furthermore, sequence of swab samples from inoculated and contact turkeys at 4 dpi indicated that mutations in the HACS were stable except for G319 which acquired one additional mutation in the HA. Whether this mutation resulted in increased virus titers in the nasal cavity in sentinel turkeys remained to be investigated. Studies showed that H9N2 HACS was stable after passaging in cell culture or in embryonated eggs^54,55^. Conversely, passaging of H9N2 in tracheal organ cultures or different birds (i.e. turkeys, quails and ducks) resulted in accumulation of mutations in the HACS, among other segments^40,56^. These different results may be explained by using different H9N2 viruses or variable host-specific selection pressure. Moreover, we detected RNA but not infectious virus in brain samples. Evidence for the extra-pulmonary spread of H9N2 in different organs in poultry has been described^48,57,58^.

Human infections caused by H9N2 have been reported^59^. Importantly, several AIV with high fatality rate in humans acquired non-HA/NA gene segments from H9N2 (e.g. H5N1, H7N9, H10N8). In addition to human hosts, H9N2 AIV has also infected mammals including pigs, dogs, cats, horses and bats^13^. Without prior adaptation, AIV H9N2 in this study succeeded in multiple-cycle replication in brain cells of pigs, cats and mice independent of trypsin, albeit to low levels. Mutation to tyrosine at P2 increased replication of WSN/H1N1 in the brain of mice^39^. Virulence of these viruses remains to be studied in mammalian models, but zoonotic risk of H9N2 AIV (e.g. G319) should be monitored carefully. G319-like H9N2 viruses may infect and replicate at higher levels in the upper respiratory tract of turkeys without causing severe disease. The threat of H9N2 AIV as a zoonotic agent is neglected^60^ and there is an urgent need to reassess containment of the H9N2 AIV in poultry^61^.

In conclusion, our analysis indicated preferential substitutions of non-basic amino acids in the HACS of European-H9N2 viruses, which were different from non-European viruses. These mutations increased virus replication in *vitro* and in vivo and contributed to virus fitness in turkeys, but not in chickens. H9N2 viruses in this study, particularly G319 replicated in mammalian cells without trypsin. The findings of this study are important to better understand adaptation of H9N2 in turkeys and mammalian cells.

## Supplementary information

Supplementary Figures.

Supplementary Table S1.

Supplementary Table S2.
