# Supplementary material for: Non-basic amino acids in the hemagglutinin proteolytic cleavage site of a European H9N2 avian influenza virus modulate virulence in turkeys
Source: Sci Rep. 2020 Dec 4;10:21226. doi: 10.1038/s41598-020-78210-8 (PMC7718272; doi:10.1038/s41598-020-78210-8)
Supplement: Supplementary file 1 — Supplementary Figures. [file 41598_2020_78210_MOESM1_ESM.docx]

**Non-basic amino acids in the hemagglutinin proteolytic cleavage site of a European H9N2 avian influenza virus modulate virulence in turkeys**

Claudia Blaurock^1^, David Scheibner^1^, Maria Landmann^2^, Melina Vallbracht^1^, Reiner Ulrich^2^, Eva Böttcher-Friebertshäuser^3^, Thomas C. Mettenleiter^1^ and Elsayed M. Abdelwhab^1*^

^1^Friedrich-Loeffler-Institut, Federal Research Institute for Animal Health, Suedufer 10, 17493 5 Insel Riems-Greifswald, Germany 6

^2^Institute of Veterinary Pathology, Faculty of Veterinary Medicine, Leipzig University, Germany

^3^Institute of Virology, Philipps University Marburg, Marburg, Germany

**Supplementary Figure S1:** Cleavability of H9N2 viruses carrying single mutations in the HACS by trypsin in chicken embryo kidney (CEK) cells.


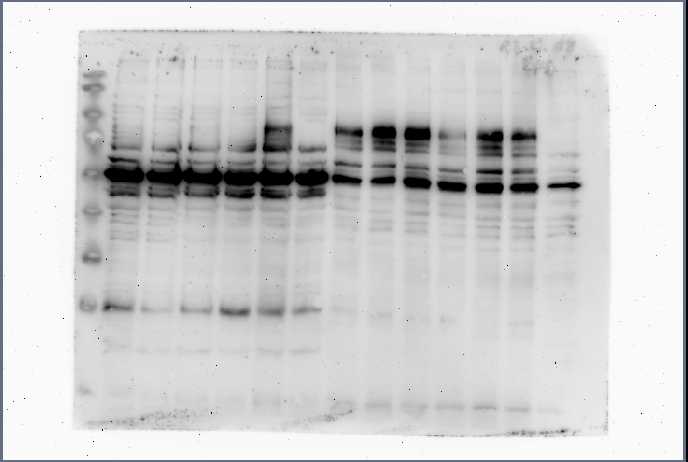


The cleavability of HA0 into HA1 and HA2 subunits was studied using Western Blot after the transfection of CEK cells with 5µg pCAGGS-HA-plasmid in the presence (+) or absence (-) of trypsin for 24 hours. Detection of the HA of all viruses was detected with serum of an infected turkey (1:100) in the current study after separation in a 12 % polyacrylamide gel. Shown, from left to right: the protein marker, G319 (+), A319 (+), N319 (+), S319 (+), D319 (+), K319 (+), G319 (-), A319 (-), N319 (-), S319 (-), D319 (-), K319 (-) and the mock control (non-infected CEK cells). For full annotation, please refer to Figure 3 panel A.

**Supplementary Figure S2:** Cleavability of H9N2 viruses carrying single mutations in the HACS by TMPRSS2 in chicken embryo kidney (CEK) cells.


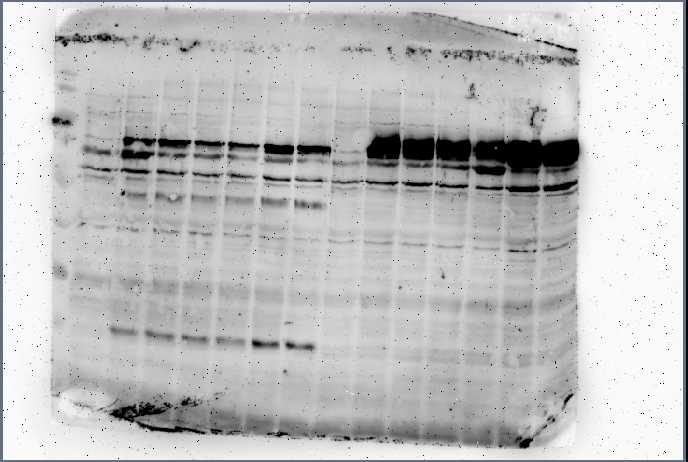


The cleavability of HA0 into HA1 and HA2 subunits was studied using Western Blot after the transfection of CEK cells with 2µg pCAGGS-HA-plasmid and with or without 500ng TMPRSS2 plasmid for 24 hours. Detection of the HA of all viruses was detected with serum of an infected turkey (1:100) in the current study after separation in a 12 % polyacrylamide gel. Shown, from left to right: protein marker, mock control (non-infected CEK cells), G319 (+TMPRSS2), A319 (+TMPRSS2), N319 (+TMPRSS2), S319 (+TMPRSS2), D319 (+TMPRSS2), K319 (+TMPRSS2), mock control (non-infected CEK cells), G319 (‑TMPRSS2), A319 (-TMPRSS2), N319 (-TMPRSS2), S319 (-TMPRSS2), D319 (-TMPRSS2) and K319 (-TMPRSS2). For full annotation, please refer to Figure 3 panel B.

**Supplementary Figure S3:** Cleavability of H9N2 viruses carrying single mutations in the HACS by MDCK cells expressing HAT.


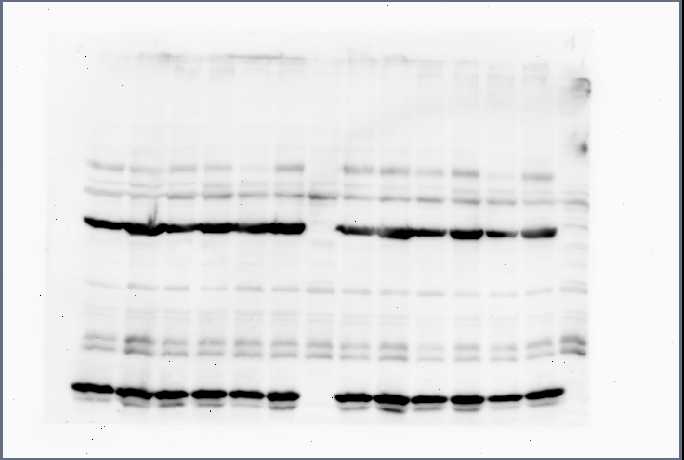


The cleavability of HA0 into HA1 and HA2 subunits was studied using Western Blot after the infection of MDCK-HAT cells with an MOI of 0.1 for 24 hours (+ 0.2 µg/ml Doxycycline). Detection of the HA of all viruses was done with serum of an infected turkey (1:100) generated in the current study after separation in a 12 % polyacrylamide gel. Shown, from left to right: protein marker, G319 (MOI = 0.1), A319 (MOI = 0.1), N319 (MOI = 0.1), S319 (MOI = 0.1), D319 (MOI = 0.1), K319 (MOI = 0.1), mock control (non-infected MDCK-HAT cells). T G319 (1 ml virus), A319 (1 ml virus), N319 (1 ml virus), S319 (1 ml virus), D319 (1 ml virus) and K319 (1 ml virus) and the mock control (non-infected MDCK-HAT cells). For full annotation, please refer to Figure 3 panel C.
