# Supplementary material for: Non-basic amino acids in the hemagglutinin proteolytic cleavage site of a European H9N2 avian influenza virus modulate virulence in turkeys
Source: Sci Rep. 2020 Dec 4;10:21226. doi: 10.1038/s41598-020-78210-8 (PMC7718272; doi:10.1038/s41598-020-78210-8)
Supplement: Supplementary file 2 — Supplementary Table S1. [file 41598_2020_78210_MOESM2_ESM.docx]

**Non-basic amino acids in the hemagglutinin proteolytic cleavage site of a European H9N2 avian influenza virus modulate virulence in turkeys**

Claudia Blaurock^1^, David Scheibner^1^, Maria Landmann^2^, Melina Vallbracht^1^, Reiner Ulrich^2^, Eva Böttcher-Friebertshäuser^3^, Thomas C. Mettenleiter^1^ and Elsayed M. Abdelwhab^1*^

^1^Friedrich-Loeffler-Institut, Federal Research Institute for Animal Health, Suedufer 10, 17493 Insel Riems-Greifswald, Germany

^2^Institute of Veterinary Pathology, Faculty of Veterinary Medicine, Leipzig University, Germany

^3^Institute of Virology, Philipps University Marburg, Marburg, Germany

**Supplementary Table S1:** **Prevalence of cleavage site motifs of H9N2 in different species**

|  | Motif  (P1/P1´) | European H9N2 | | | | Non-European H9N2 | | | |
| --- | --- | --- | --- | --- | --- | --- | --- | --- | --- |
|  |  | Total No. | Chicken | Turkey | Other* | Total No. | Chicken | Turkey | Other |
| 1 | AS**G**R/G | 2 | 0 | 0 | 2 | 13 | 6 | 0 | 7 |
| 2 | AS**A**R/G | 6 | 0 | 5 | 1 | 0 | 0 | 0 | 0 |
| 3 | AS**N**R/G | 11 | 0 | 8 | 3 | 3 | 0 | 0 | 3 |
| 4 | RS**S**R/G | 9 | 9 | 0 | 0 | 2384 | 1842 | 11 | 531 |
| 5 | AS**D**R/G | 39 | 0 | 0 | 39 | 70 | 1 | 1 | 68 |
| 6 | AS**K**R/G | 4 | 0 | 4 | 0 | 0 | 0 | 0 | 0 |
| 7 | RSKR/G | 3 | 3 | 0 | 0 | 6 | 0 | 5 | 1 |
| 8 | VSDR/G | 1 | 0 | 0 | 1 | 20 | 0 | 6 | 14 |
| 9 | TSNR/G | 2 | 1 | 1 | 0 | 0 | 0 | 0 | 0 |
| 10 | TSGR/G | 3 | 1 | 2 | 0 | 16 | 14 | 0 | 2 |
| 11 | ISGR/G | 1 | 0 | 1 | 0 | 2 | 0 | 0 | 2 |
| 12 | ISDR/G | 1 | 1 | 0 | 0 | 0 | 0 | 0 | 0 |
| 13 | RSGR/G |  |  |  |  | 3 | 2 | 1 | 0 |
| 14 | RSNR/G |  |  |  |  | 35 | 21 | 0 | 14 |
| 15 | ASDK/G |  |  |  |  | 1 | 0 | 0 | 1 |
| 16 | VSNR/G |  |  |  |  | 7 | 0 | 0 | 7 |
| 17 | VSGR/G |  |  |  |  | 7 | 0 | 1 | 6 |
| 18 | VSSR/G |  |  |  |  | 9 | 2 | 6 | 1 |
| 19 | ASYR/G |  |  |  |  | 7 | 6 | 0 | 1 |
| 20 | ISNR/G |  |  |  |  | 3 | 0 | 0 | 3 |
| 21 | KSSR/G |  |  |  |  | 172 | 135 | 0 | 37 |
| 22 | ISSR/G |  |  |  |  | 1 | 1 | 0 | 0 |
| 23 | KASR/G |  |  |  |  | 2 | 1 | 0 | 1 |
| 24 | RYSR/G |  |  |  |  | 2 | 2 | 0 | 0 |
| 25 | RASR/G |  |  |  |  | 21 | 4 | 0 | 17 |
| 26 | RLSR/G |  |  |  |  | 13 | 11 | 0 | 2 |
| 27 | RSRR/G |  |  |  |  | 7 | 4 | 0 | 3 |
| 28 | GSSR/G |  |  |  |  | 3 | 3 | 0 | 0 |
| 29 | RPSR/G |  |  |  |  | 2 | 1 | 0 | 1 |
| 30 | RFSR/G |  |  |  |  | 9 | 8 | 0 | 1 |
| 31 | RSTR/G |  |  |  |  | 2 | 2 | 0 | 0 |
| 32 | RSSK/G |  |  |  |  | 4 | 3 | 0 | 1 |
| 33 | RCSR/G |  |  |  |  | 1 | 0 | 0 | 1 |
| 34 | RSIR/G |  |  |  |  | 1 | 0 | 0 | 1 |
| 35 | KSKR/G |  |  |  |  | 96 | 41 | 0 | 55 |
| 36 | RSNK/G |  |  |  |  | 1 | 1 | 0 | 0 |
|  | Total | 82 | 15 | 21 | 46 | 2926§ | 2111 | 31 | 781 |

* “Other” refers to e.g. ducks, mallards, teals, wigeons, pheasants, ostriches, swans, pigeons or environment.

§ Three sequences from chickens (2 from Iran in 2007 and 1 from Egypt in 2013) had RSNR/**R**, RSNK/**R** and KSSR/**A** motifs assuming wrong or unusual sequences in the HA2 (i.e. underlined R or A).
