# Supplementary material for: Non-basic amino acids in the hemagglutinin proteolytic cleavage site of a European H9N2 avian influenza virus modulate virulence in turkeys
Source: Sci Rep. 2020 Dec 4;10:21226. doi: 10.1038/s41598-020-78210-8 (PMC7718272; doi:10.1038/s41598-020-78210-8)
Supplement: Supplementary file 3 — Supplementary Table S2. [file 41598_2020_78210_MOESM3_ESM.docx]

**Non-basic amino acids in the hemagglutinin proteolytic cleavage site of a European H9N2 avian influenza virus modulate virulence in turkeys**

Claudia Blaurock^1^, David Scheibner^1^, Maria Landmann^2^, Melina Vallbracht^1^, Reiner Ulrich^2^, Eva Böttcher-Friebertshäuser^3^, Thomas C. Mettenleiter^1^ and Elsayed M. Abdelwhab^1*^

^1^Friedrich-Loeffler-Institut, Federal Research Institute for Animal Health, Suedufer 10, 17493 Insel Riems-Greifswald, Germany

^2^Institute of Veterinary Pathology, Faculty of Veterinary Medicine, Leipzig University, Germany

^3^Institute of Virology, Philipps University Marburg, Marburg, Germany

**Supplementary Table S2: Clinical examination of chickens after oculonasal challenge**

|  |  | | Inoculated chickens | | | Contact chickens | | |
| --- | --- | --- | --- | --- | --- | --- | --- | --- |
|  | PI | Morbidity | | Shedding | Seroconversion | Morbidity | Shedding | Seroconversion |
| K319 |  | 0/10* | | 8/10 | 7/7 | 0/5 | 0/5 | 0/5 |
| K319G |  | 0/10 | | 7/10 | 7/7 | 0/5 | 0/5 | 0/5 |
| K319A |  | 0/10 | | 8/10 | 7/7 | 0/5 | 0/5 | 0/5 |
| K319N |  | 0/10 | | 5/10 | 7/7 | 0/5 | 0/5 | 0/5 |
| K319S |  | 0/10 | | 9/10 | 7/7 | 0/5 | 0/5 | 0/5 |
| K319D |  | 0/10 | | 4/10 | 7/7 | 0/5 | 0/5 | 0/5 |

* Number of positive birds/total examined

Chickens were challenged with 10^5.7^ pfu/bird and 1 dpi 5 birds were added to assess transmission. At 4 dpi, 3 directly-inoculated chickens were euthanized to assess virus distribution and lesions in different organs. Seroconversion (number of positive birds / total examined) was tested at 10 dpi using ELISA. Clinical scoring was conducted as recommended by the OIE on a scale 0 to 3: 0= apparently healthy, 1= birds showed 1 clinical sign (ruffled feather, respiratory disorders, diarrhea), 2 = birds showed more than 1 clinical signs and 3 = dead birds. The pathogenicity index (PI) is the mean of all clinical scores for all inoculated birds in 10 day-observation period. All birds remained healthy. Clinical examination was done blindly by two veterinarians. Shedding was determined by RT-qPCR.
